# Application of Adult and Pluripotent Stem Cells in Interstitial Cystitis/Bladder Pain Syndrome Therapy: Methods and Perspectives

**DOI:** 10.3390/jcm9030766

**Published:** 2020-03-12

**Authors:** Ahmed Abdal Dayem, Kyeongseok Kim, Soo Bin Lee, Aram Kim, Ssang-Goo Cho

**Affiliations:** 1 Department of Stem Cell & Regenerative Biotechnology and Incurable Disease Animal Model and Stem Cell Institute (IDASI), Konkuk University, 120 Neungdong-ro, Gwangjin-gu, Seoul 05029, Korea; ahmed_morsy86@yahoo.com (A.A.D.); proproggs@naver.com (K.K.); soobineey@naver.com (S.B.L.); 2 Department of Urology, Konkuk University Medical Center, Konkuk University School of Medicine, Seoul 05029, Korea

**Keywords:** IC/BPS, PSCs, ASCs, therapy, regeneration, urothelium, differentiation, transplantation, bladder

## Abstract

Interstitial cystitis/bladder pain syndrome (IC/BPS) is a multifactorial, chronic disease without definite etiology characterized by bladder-related pelvic pain. IC/BPS is associated with pain that negatively affects the quality of life. There are various therapeutic approaches against IC/BPS. However, no efficient therapeutic agent against IC/BPS has been discovered yet. Urothelium dysfunction is one of the key factors of IC/BPS-related pathogenicity. Stem cells, including adult stem cells (ASCs) and pluripotent stem cells (PSCs), such as embryonic stem cells (ESCs) and induced PSCs (iPSCs), possess the abilities of self-renewal, proliferation, and differentiation into various cell types, including urothelial and other bladder cells. Therefore, stem cells are considered robust candidates for bladder regeneration. This review provides a brief overview of the etiology, pathophysiology, diagnosis, and treatment of IC/BPS as well as a summary of ASCs and PSCs. The potential of ASCs and PSCs in bladder regeneration via differentiation into bladder cells or direct transplantation into the bladder and the possible applications in IC/BPS therapy are described in detail. A better understanding of current studies on stem cells and bladder regeneration will allow further improvement in the approaches of stem cell applications for highly efficient IC/BPS therapy.

## 1. Introduction

Interstitial cystitis/bladder pain syndrome (IC/BPS) is a chronic disease, and patients with IC/BPS without any underlying infections struggle with symptoms of frequent and urgent urination, pelvic pain, and nocturia [1]. The pain is especially aggravated during bladder filling. In addition, the pain associated with the disease results in other serious consequences such as stress, depression, poor sexual function, and anxiety, which negatively influence the quality of the patient’s professional and social life [2,3,4]. In 2002, the name IC was changed to IC/BPS by the International Continence Society to define the condition as the complaint of suprapubic pain associated with urine storage and the frequent and urgent need to urinate in the absence of a urinary tract infection (UTI) [5]. The cost of the medical management of IC/BPS is estimated to be more than $11,000 per patient annually. Moreover, due to the chronic nature of the disease, the expenses are generally incurred for several years [6,7,8]. Furthermore, the need for narcotic medication as a line of treatment adds to the economic burden of caring for a patient with IC/BPS [9].

Urothelium, which is a unique type of epithelial tissue that lines the wall of the urinary tract, including the proximal urethra and the urinary bladder, plays an essential role as a barrier against pathogens, toxins, and wastes from the urine [6,10]. Notably, the urothelium constitutes the urine-blood barrier and is supported by thick pseudostratified transitional epithelium (multi-layered) and the asymmetric and fully differentiated superficial membrane (umbrella) composed of complexes of uroplakins (UPs) [11,12,13]. The permeability of the urothelium is attributed to these unique proteins, known as the UPs, which comprise four subtypes: UP1a, UP1b, UP2, and UP3. These aggregate to produce crystalline plaques on the surface of the bladder lumen [13,14]. Proliferation is a response by the urothelium toward any injury of the superficial layer to restore the urine-blood barrier [15,16]. The damaged bladder urothelium is the main factor in the IC/BPS-associated crippled barrier function that results in bladder pain during urine storage [17]. Defects in the urothelium barrier result in the leakage of urine solutes, such as potassium, into the suburothelium or the lamina propria, which leads to nerve depolarization and consequent inflammation-associated injuries, urgency, and pain in IC/BPS [18,19,20]. Notably, IC/BPS is implicated in the sensitization of the central nervous system (CNS) and afferent nerves, which leads to hyperalgesia and allodynia [21,22].

In 1915, Guy Hunner first discovered the ulcers, which was detected by cystoscopy in 10–15% of IC/BPS patients [23]. These ulcers were later named Hunner’s lesions due to the formation of ulcerative areas in the bladder urothelium [24]. Specifically, Hunner’s lesions are red-colored mucosal regions in which tiny vessels are directed toward a central scar [25]. IC/BPS can be broadly classified as an ulcerative or non-ulcerative type based on the presence or absence of Hunner’s lesions, respectively. In addition, Hunner’s lesions occur in approximately 50% of IC/BPS patients [25,26]. IC/BPS is more prevalent in women than in men with a female to male ratio of 10:1 [27]. Nevertheless, recent reports demonstrate a higher prevalence in men than that reported previously [28,29]. IC/BPS commonly occurs in middle-aged to old-aged females with the prevalence of more than 2% [17,30]. In the United States, around 2.7–6.5% of females are diagnosed with symptoms of IC/BPS [31,32].

Various etiological factors are implicated in the pathogenicity of IC/BPS, such as inflammation, neuropathy, injury to the urothelium and disturbance in its function, damage of the glycosaminoglycan (GAG) layer, mast cell activation, and autoimmune disorders [33,34,35,36]. Additionally, stress or chronic stress strongly contributes to the occurrence, aggravation, and pathogenicity of IC/BPS [37,38,39,40]. There are a wide range of therapeutic approaches that target the previously mentioned factors. However, these therapeutic approaches do not afford complete recovery, and the recurrence of symptoms is reported even after treatment completion.

The lack of suitable tissue sources and senescence-associated long-term primary cultures of bladder cells is the main hurdle in bladder tissue restoration [41,42]. For instance, the application of gastrointestinal tract-derived tissue for bladder restoration has many challenges, including complications associated with tissues variations, tumorgenicity, intermittent infection, urinary stones, and metabolic disorders [43,44]. Therefore, alternative therapeutic approaches, such as stem cell-based therapies, should be considered. The promise of stem cell therapy as an approach for IC/BPS therapy is attributed to the ability of these cells to differentiate into multiple lineages, including bladder cells [45,46]. In this review, we provide a brief overview of the causes and pathogenicity of IC/BPS as well as the conventional therapeutic approaches against IC/BPS. Moreover, recent advances in the applications of adult stem cells (ASCs) as well as pluripotent stem cells (PSCs) in bladder regeneration and IC/BPS therapy are described in detail along with a summary of their mechanisms and limitations. We believe that a proper understanding of the manner in which stem cells work in bladder disease therapy as well as the limitations of stem cells will provide clues to researchers and clinicians to further improve the use of stem cells as potent therapeutic tools against IC/BPS.

## 2. Etiology and Pathophysiology of IC/BPS

**Figure 1:**
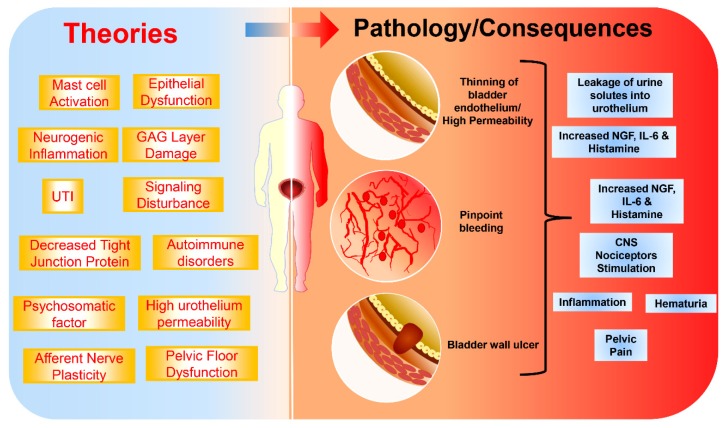
Pathophysiology of interstitial cystitis/bladder pain syndrome (IC/BPS).

The definite causes of IC/BPS are not yet clearly known [47]. However, many theories have emerged to explain the pathogenicity of IC/BPS. There are a wide range of factors implicated in IC/BPS such as bladder epithelial dysfunction, mast cell activation, neurogenic inflammation, tight junction protein downregulation, afferent nerve plasticity, bacterial infection, urothelial signaling disturbances, and psychosomatic factors (Figure 1) [48,49,50]. UTIs, which is one of the aggravating causes of IC/BPS, occur at a young age and lead to IC/BPS in adulthood [51].

The Hunner’s lesion type of IC/BPS is attributed to factors secreted by mast cells, whereas, in non-ulcerative type IC/BPS, patients have a normal number of mast cells [52,53]. The high permeability of the bladder urothelium is among the causes of IC/BPS, which is associated with damage to the superficial layer of the extracellular matrix of the urothelial GAG layer and its components, such as chondroitin sulfate, hyaluronic acid, and heparin sulfate [54]. Such damage leads to a fragile urothelium. The consequent diffusion of potassium, which infiltrates into the nerves, causes pain during voiding (urination) [20,55].

Heparin-binding epidermal growth factor (HB-EGF)-like GF [56], connexin 43, and 45 [57], nitric oxide (NO) [58], anti-proliferative factor [59], and disturbed tight junctions, including Zona Occludens 1 (ZO-1) [60], are also key mediators in the dysfunction of urothelial permeability. Consequently, a bladder with a defective barrier function allows the passage of toxic byproducts into the suburothelium layer and then activates the inflammatory responses and the afferent pathways, which ultimately contribute to pain and urgency [61]. Notably, stressors can be important factors in the aggravation of IC/BPS symptoms [37,62]. Persistent stimulation of CNS nociceptors as well as synaptic plasticity are the main causes of continual pain [63].

The increased number of mast cells in the bladder wall of IC/BPS patients is implicated in severe inflammation, allergic reactions, and increased bladder sensitization [64,65,66,67]. The activated mast cells are involved in the upregulation of nerve GF (NGF), interleukin-6 (IL-6), histamine/methylhistamine, and tryptase, which possess nociceptive, pro-inflammatory, and vasoactive actions that are crucial in the pathogenicity of IC/BPS [68,69]. When the bladder is exposed to insult, stress, and acetylcholine stimulation as well as hormonal disorders, the mast cells are activated and histamine is released, which consequently lead to neuro-inflammation, pain, muscle contraction, and urination frequency and urgency [70,71]. Therefore, the clinical application of mast cell inhibitors in IC/BPS therapy has been reported [72,73].

Autoimmunity is also one of the chief factors in the pathogenicity of IC/BPS. In this regard, patients with known autoimmune diseases are similar to IC/BPS patients in age and sex [34,74]. Moreover, the correlation between the occurrence of clinical IC/BPS and autoimmune disease has been previously demonstrated [34]. The presence of autoantibodies in IC/BPS patients is a secondary symptom of autoimmune disease. The production of autoantibodies and autoreactive B and T cells is one of the mechanisms of autoimmunity [75]. These autoantibodies have a negative impact on the urothelium, smooth muscles, and connective tissues of the urinary bladder [34]. For instance, in autoimmune diseases, such as Sjögren’s syndrome, the production of autoantibodies to the muscarinic receptor (M3R) in lacrimal and salivary glands has been shown [34]. Similarly, M3R is involved in the contractile function of the bladder detrusor muscle, which supports the auto-immunity-IC/BPS correlation [34]. Recently, a nation-wide study demonstrated the occurrence of IC/BPS in patients with primary Sjögren’s syndrome [76]. Thus, there is a robust correlation between autoimmune diseases and the pathogenicity of IC/BPS, which is shown by the presence of autoantibodies and chronic inflammation.

IC/BPS is also correlated with chronic pain-associated diseases including fibromyalgia, irritable bowel syndrome, vulvodynia, endometriosis, and chronic fatigue syndrome [47,77]. Moreover, environmental factors, such as the ingestion of certain foods that induce allergy, could aggravate IC/BPS symptoms [78,79]. In 2011, Bassaly et al. reported the occurrence of myofascial pain in 78.3% of IC/BPS patients with a minimal of one trigger point [80]. Clinical examination of the abdominal/pelvic floor muscle allows the appropriate recognition of the focal myofascial-causing factors helpful in improving IC/BPS therapy.

## 3. IC/BPS Diagnosis

The American Urological Association (AUA), the European Society for the Study of IC (ESSIC), and the Society of Interstitial Cystitis of Japan (SICJ) are global societies that issue guidelines for the diagnosis and treatment of IC/BPS [81,82,83,84]. The following symptoms are indicative of IC/BPS: dyspareunia, prostatitis (male) or vaginitis (female), pelvic pain, frequency, urgency, nocturia, and urinary tract infection [85,86,87]. Due to the lack of IC symptom specificity, misdiagnoses or late diagnoses commonly occur [55]. Generally, the clinical examination involves patient history (onset, duration, lifestyle-related inducing factors, and symptoms), physical examination, urine analysis, questionnaires, and cystoscopy. In particular, a pelvic examination is essential to exclude other disorders, such as pelvic floor dysfunction, vaginitis, urethral diverticula, and vulvar lesions [85]. Notably, during IC/BPS diagnosis, overlapping diseases that share similar symptoms with IC/BPS should be carefully considered [88]. In addition, clinical examination of the muscle strength of the pelvic floor is essential for the diagnosis of IC/BPS [81,89].

The patient’s dietary regimen should be thoroughly screened to determine any dietary factors that may aggravate or relieve the symptoms of IC/BPS [71,81]. The O’Leary-Sant (OLS) questionnaire is commonly administered by clinicians for the diagnosis of IC/BPS since it contains a symptom index for pain and urine urgency [90]. In addition, OLS is helpful for measuring the degree of improvement after therapy [70,91].

A cystoscopy examination to monitor the histological features of Hunner’s lesions or glomerulations is helpful for the diagnosis of IC/BPS [83,88]. A burst of Hunner’s lesions occurs upon bladder distension and is accompanied by oozing blood and bullous edema [83,88]. However, recent literature concluded a lack of correlation between glomerulations and an IC/BPS diagnosis [88,92]. More details on the diagnosis of IC/BPS are provided elsewhere [48,93,94,95].

## 4. IC/BPS Therapeutic Approaches

The main goals of IC/BPS therapy are to mitigate and treat the consequent symptoms (stress and anxiety), repair impaired bladder function, treat inflammation, and restore neuronal dysfunction [96]. With regard to IC/BPS therapy, several guidelines have been recommended by various international societies [24,81,93,97]. The AUA guidelines provide a stepwise therapeutic approach in which conservative therapies are first attempted. If the severity of the symptoms is not alleviated, more strenuous therapeutic approaches are then carried out [82,98].

The first line therapeutic approach includes patient education to alleviate symptoms associated with daily behavioral habits or lifestyles, such as fluid and food intake, type of clothing worn, sexual activity, and habits that promote stress and pain [93]. For instance, avoiding fluids and diets that stimulate the bladder, such as potassium-rich food and food with a high acid content, including chocolate, tomatoes, citrus, alcohol, and coffee, is essential to reduce symptom severity [85]. Moreover, increasing patient awareness of normal bladder function and educating patients on the pros and cons of using currently available therapeutic options or a combination of various drugs is necessary to alleviate the symptoms [93].

Second-line therapies include physical treatment approaches, oral administration of therapeutic agents, such as hydroxyzine, cimetidine, amitriptyline, and pentosan polysulfate sodium (PPS), and the intravesicular application of heparin, lidocaine, chondroitin sulfate, steroids (triamcinolone), botulinum toxin, or dimethylsulfoxide (DMSO) [53,81,99]. The intravesicular route of administration ensures the delivery of a high concentration of drugs to the bladder tissue with minimal side effects. However, the frequent catheterization associated with intravesicular instillation can lead to pain and occasional UTIs [27,100]. The therapeutic agents for IC/BPS and the routes of administration have been reviewed elsewhere [17,101]. Myofascial physical therapy (MPT) and global therapeutic massage (GTM) can be performed depending on the patient status [102]. DMSO is only approved by Food and Drug Administration (FDA) for intravesicular administration [98]. However, if a patient does not experience relief from symptoms after the application of such therapeutic agents, surgical intervention is needed. For instance, cystectomy leads to long-lasting alleviation of symptoms. It was reported that 74% of patients subjected to cystectomy showed no pain symptoms for 66 months [103]. Nonetheless, the surgical approach does not guarantee pain relief [17]. Narcotics, non-narcotic compounds, non-steroidal anti-inflammatory drugs (NSAIDs), and urinary analgesics (methenamine and phenazopyridine) can be used as pain control agents [98]. Simultaneously, the side effects of the pain-controlling agents should be meticulously considered. Some reports are showing the therapeutic capacities of the natural compounds, such as curcuminoids, flavonoids, aloe vera extract, herbal medicine, and other natural alternative medicines against the IC-related pathological changes [104,105,106,107,108]. These natural compounds possess minimal side effects compared to the chemical agents and, therefore, their clinical application in IC/BPS therapy should be considered in the future. The therapeutic approaches against IC/BPS have been reviewed elsewhere [7,101,109,110]. Although strategies for IC/BPS therapy exist, such as those described above, there is no IC/BPS treatment available that can ensure long-term efficacy and cure [82]. Therefore, stem cell therapy could be a promising long-term and efficacious therapeutic tool against IC/BPS.

## 5. Overview of ASCs and PSCs

**Figure 2:**
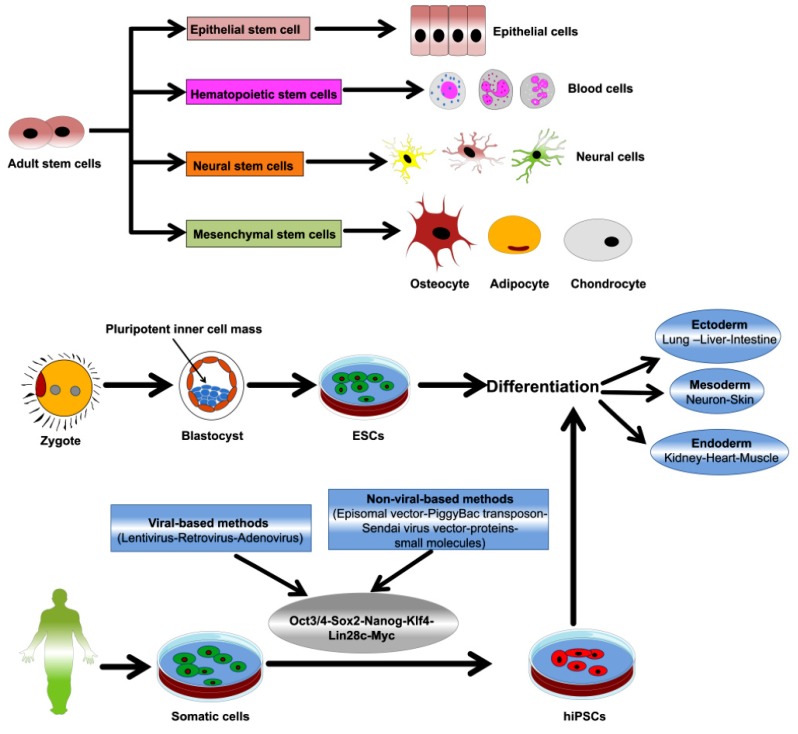
Representative figure illustrating stem cell types and their differentiation abilities. Reproduced from an article by Abdal Dayem et al. 2018 [143], an open access article distributed under the terms and conditions of the Creative Commons Attribution (CC BY) license (http://creativecommons.org/licenses/by/4.0/).

Stem cells have unique abilities of self-renewal, proliferation, and differentiation. Therefore, they represent a robust tool in tissue regeneration and could be an alternative to other therapeutic approaches [111,112]. Stem cells are broadly classified as ASCs, embryonic stem cells (ESCs), and induced pluripotent stem cells (iPSCs) (Figure 2). ESCs are derived from the inner cell mass (ICM) of early embryos and can differentiate into three types of germ layer cells in vitro and in vivo [113]. PSCs (ESCS and iPSCs) can be cultured to remain in the undifferentiated state for a minimum of 80 passages and 200 population doublings [114,115,116]. Beyond ethical issues, biological issues, such as immune activation, make the application of ESCs in tissue regeneration less feasible. Accordingly, iPSC technology could be a powerful alternative to ESCs as it circumvents the concerns that arise from using embryos as in ESCs. iPSCs are generated by reprogramming somatic cells, using viral and non-viral induction methods, to express specific transcription factors, including Oct3/4, Sox2, Klf4, Nanog, c-myc, and Lin28 [117]. The resulting iPSCs possess the pluripotency, proliferation capacity, gene expression patterns, differentiation capacity, and epigenetic profiles shown by ESCs [118]. In contrast, considerable research has demonstrated the regenerative abilities of ASCs, such as mesenchymal stem cells (MSCs) and hematopoietic stem cells (HSCs) in various disorders. Notably, ASCs preclude the ethical concerns, histocompatibility issues, and tumorigenicity that arise with the use of PSCs [119,120,121]. MSCs are an important class of ASCs that can be isolated from several tissues and organs of adults and neonates, such as bone marrow, adipose tissue, urine, placenta, and an umbilical cord. Furthermore, MSCs have been studied for a long time and have been applied in various experimental studies and preclinical investigations [122,123,124]. MSCs possess the features of real stem cells while retaining the characteristics of the progenitor cells of the tissue source (heterogenicity). Therefore, the term MSCs is being currently changed to “mesenchymal stromal cells” and “medicinal signaling cells” [125,126]. The therapeutic potential of MSCs is attributed to its properties such as paracrine function, immunomodulation, homing, and migration [127,128,129,130]. The contribution of MSCs in the cure of various chronic and acute diseases including heart disease, spinal cord injury, lung disease, musculoskeletal disorders, inflammatory disease, and autoimmune disorders has been demonstrated previously [131,132,133,134]. There are more than 800 registered clinical trials (www.clinicaltrials.gov) investigating the therapeutic capacities of MSCs in various diseases, including rheumatoid arthritis, degenerative diseases of the bone and cartilage, wound healing, neurological disorders, diabetes, multiple sclerosis, and cardiovascular diseases [135,136]. Hence, stem cells could also be a potent therapeutic platform against bladder diseases [36,137]. Notably, the therapeutic function and characteristics of MSCs differ based on the tissue of origin and the donor [138,139,140,141,142]. In the following sections, we provide a detailed description of the possible applications of ASCs and PSCs in IC/BPS therapy and discuss their limitations and prospects.

## 6. ACSs and IC/BPS Therapy

The contribution of ASCs in the treatment of IC/BPs is attributed to their ability to differentiate into bladder cells, such as urothelial cells and smooth muscle cells (SMCs), in the urothelium or their survivability when transplanted directly into the injured bladder.

### 6.1. Differentiation into Bladder Cells

The urinary bladder, which is a hollow organ, is composed of tissue of the urothelium (from the endoderm) and SMCs (from the mesoderm). The interactions between these components are crucial for preserving the structural integrity as well as the functionality of the bladder. Below, we describe the approaches for the differentiation of ASCs into bladder cells, including the urothelium and SMCs.

In 2010, Tian et al. demonstrated the differentiation of human bone marrow-derived MSCs (hBM-MSCs) into urothelium and SMCs using conditioned media (CM) or a co-culture system [144]. hBM-MSCs were co-cultured with patient-derived bladder urothelial cells or cultured in CM derived from bladder urothelial cell cultures. For the co-culture, bladder urothelial cells were placed in the upper chamber and hBM-MSCs were cultured in the lower chamber of a 10-cm Transwell plate separated by a 0.4-µm membrane barrier. High expression of the urothelium-associated genes and proteins, such as UP1a, cytokeratin (CK)-7, and CK-13, proved that urothelium differentiation was successful [144]. The differentiation of hBM-MSCs into urothelium and SMCs using bladder cells cultured in CM was superior to that using the co-culture system. This superior effect is attributed to the high content of cytokine GF, such as a platelet-derived GF-homodimer polypeptide of B chain (PDGF-BB), transforming GF-βeta (TGF-β), and vascular endothelial GF (VEGF) in the prepared CM.

In 2011, Ning et al. induced urothelium differentiation from BM-MSCs via co-culture with neonatal urothelial cells derived from isolated human fetal urinary bladders for two weeks [145]. The BM-MSCs differentiated into epithelial cells showed upregulation of urothelium-related keratin levels.

In 2013, Wu et al. reported that human umbilical cord-derived mesenchymal stromal cells (hUC-MSCs) differentiated into urothelium upon culturing with urothelial cell-derived CM containing 20 ng/mL exogenous EGF [146]. The expression of UPs and CK began one week after the initiation of differentiation, and significant increases in their expression were detected after two weeks.

Urine-derived stem cells (USCs), which is easily isolated from the urine (from the upper urinary tract) without any invasive surgical intervention, have stem cell properties, such as clonogenicity, multi-lineage differentiation capacities, high growth and proliferation potential, paracrine effects, and immunomodulatory functions [147,148,149]. Since USCs are easily obtained from urine excreted from the upper urinary tract, they are considered a suitable and safe source of cells for bladder regeneration [150]. Notably, bladder augmentation using intestinal segments resulted in the emergence of malignancy (adenocarcinoma), which is ascribed to the alteration of the intestinal mucosa after long-term exposure to urine [151,152]. Therefore, USCs could be a potent cell source for bladder regeneration. Using various induction methods, Wan et al. induced the differentiation of USCs isolated from the urine of healthy individuals into urothelium [153]. The authors proved that the exposure of USCs to urothelial cell-derived CM resulted in a differentiation capacity that was superior to that shown by the other methods. The differentiation was confirmed by the increased expression of the urothelial markers UP II and UP1a, tight junction markers known as cingulin, E-cadherin, ZO-1, and ZO-2, and epithelial markers called CK-AE1/AE3 and CK-20. Additionally, the barrier function of the derived urothelium was confirmed by the marked decrease in permeability as detected using a fluorescent dye [153]. The in vivo therapeutic application of this derived urothelium needs to be verified and further studied in an IC/BPS model. Sun et al. recently formulated a novel protocol for the differentiation of human USCs (hUSCs) into the interstitial cells of Cajal-like cells (ICC-LCs) [154]. ICC-LCs play an important function as pacemaker cells that regulate the spontaneous contraction of the bladder [155,156]. For differentiation into ICC-LCs, hUSCs were transfected with lentiviral vectors that encode the stem cell factor (SCF), c-kit, the 5-azacytidine-induced 2 (AZI2) gene, and hyperpolarization-activated cyclic nucleotide gated potassium channel 4 (HCN4) [154]. In this study, the low efficiency of differentiation did not allow in vivo validation. Thus, further validation studies are needed to improve the differentiation efficiency of hUSCs into ICC-LCs in vivo.

In 2014, Kang et al. enhanced the differentiation of human amniotic fluid-derived stem cells (hAFSCs) into urothelium via co-culture with bladder cancer-derived CM for two weeks [157]. The differentiated hAFSCs showed significantly decreased mRNA and protein levels of pluripotency markers, whereas they showed increased expression of urothelial cell-related markers, UPII, CK-8, and fibroblast GF (FGF) 10. Interestingly, increases in muscle-specific kinase (MuSK), Tyrosine kinase with immunoglobulin-like and EGF-like domains 1 (Tie-1), and Ephrin type-A4 (EphA4) receptor tyrosine kinases and decreases in EphA7 and FGF receptor 1 (FGF R1) kinases were detected [157]. Moreover, Chung et al. induced hAFSCs to differentiate into urothelium via co-culture with immortalized bladder cancer cell lines (LD605, LD611, and LD627), which is mediated by FGF10 signaling [158]. The FGF family plays crucial roles in cell growth, morphogenesis, and embryogenesis. In particular, FGF10 acts in a paracrine manner as a mitogen to promote the proliferation of the bladder epithelium. Therefore, FGF10 plays a crucial role in the regeneration of the damaged bladder epithelium [159]. In this study, co-culture was performed indirectly for up to one month using pore-sized membrane tissue culture inserts [158]. The induction of urothelium differentiation was shown by the high expression of UP II, UP III, and CK-8, which was detected by a reverse transcription polymerase chain reaction (RT-PCR), Western blot, and immunostaining. Knockdown of FGF10 expression in the bladder cancer cells abrogated the induction of urothelium differentiation in AFSCs. Additionally, the authors detected no induction of urothelium differentiation upon inhibition of downstream signaling in the bladder cancer cells using U0126, which is a mitogen-activated protein kinase kinase (MEK) inhibitor [158]. Notably, the safety of the differentiated AFSCs after co-culturing with bladder cancer cells was monitored for tumorigenicity using a soft agar colony assay, which showed negative results [158]. However, in vivo validation studies of the differentiated cells in the regeneration of damaged bladder tissue are still needed.

In 2016, Song et al. successfully induced the differentiation of dental pulp-derived stem cells (DPSCs) into bladder SMCs in culture using bladder SMC-derived CM plus TGF-β1 [160]. Thereafter, the authors confirmed the upregulation of SMC-related genes and proteins, namely alpha smooth muscle actin (α-SMA), desmin, and calponin. In 2019, Jiang et al. proved the crucial role of the canonical Wnt-GSK3β/β-catenin pathway in the differentiation of DPSCs into bladder SMCs [161]. However, these findings need to be verified in IC/BPS animal models.

In 2012, Shi et al. reported the transdifferentiation of human adipose-derived stem cells (hADSCs) into urothelial cells after exposure to urothelium-derived CM [162]. Two weeks after culturing in urothelium-derived CM, approximately 25–50% of hADSCs showed a change in their phenotype and exhibited a polygonal epithelium-like morphology. Besides the altered morphology, the differentiated cells showed increased expression levels of urothelium-related markers, namely UP2 and CK-18, and decreased expression levels of vimentin (a mesenchymal marker). The authors detected high levels of PDGF-BB and VEGF in the CM. However, the therapeutic efficacy of hADSC-derived urothelium needs to be verified in further studies in vivo using IC/BPS animal models. Overall, the potential of ASCs to differentiate into bladder urothelium is promising for the regeneration of the damaged bladder.

### 6.2. Direct Transplantation

**Figure 3:**
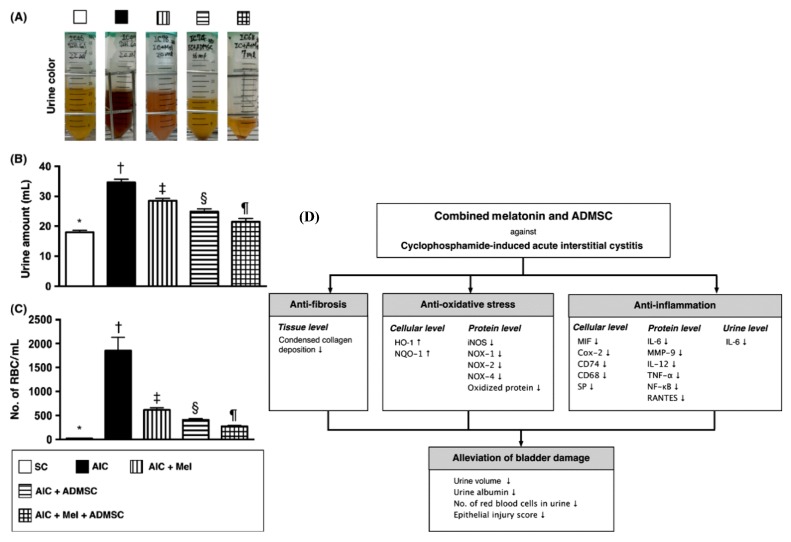
Effect and mechanisms of combination therapy with adipose-derived mesenchymal stem cells (AD-MSCs) and melatonin in cyclophosphamide (CYP)-induced acute interstitial cystitis (AIC). (**A**) The AIC group showed the highest urine turbidity and hematuria compared to the other groups. In contrast, the group treated with the combination of AD-MSCs and melatonin showed markedly lower urine turbidity than the groups treated with AD-MSCs or melatonin alone. This test was carried out for 72 h after AIC induction. (**B**) The urine amount was recorded on the last day of the 72-h period after AIC induction. (**C**) Number of red blood cells (RBCs) per mL of urine. (**D**) The mechanisms underlying the effects of the combination of AD-MSCs and melatonin in CYP-induced AIC therapy. These figures were reproduced from articles by Chen et al. 2014 [163] with permission from John Wiley and Sons. TGF-β1, transforming growth factor-beta 1. EB, embryoid bodies. HEMA, hydroxyethyl methacrylate. HO, heme oxygenase. iNOS, inducible nitric oxide synthase. NOX, NADPH oxidase. NF-*κ*B, nuclear factor kappa-light-chain-enhancer of activated B cells. NQO, NAD(P)H quinone oxidoreductase. MMP-9, matrix metalloproteinases-9. IL, interleukin. AIC, acute interstitial cystitis. TNF-*α,* tumor necrosis factor-*α*. MIF, macrophage migration inhibitory factor. SP, substance P. RBCs, red blood cells. Down arrow, downregulation. Up arrow, upregulation.

In 2014, Chen et al. demonstrated the potential of combining adipose-derived MSCs (AD-MSCs) and melatonin in acute IC (AIC) in rats [163]. The induction of AIC was performed by an intraperitoneal (i.p.) injection of cyclophosphamide (CYP) at 150 mg/kg. This combination therapy with AD-MSCs and melatonin caused a significant improvement in CYP-induced AIC symptoms, including voiding and hematuria, compared to treatment with either AD-MSCs or melatonin alone (Figure 3). Moreover, the combination therapy significantly downregulated the protein expression of the inflammatory markers (IL-6, IL-12, nuclear factor kappa B (NF-κB), tumor necrosis factor alpha (TNF-α), RANTES, inducible NO synthase (iNOS), Matrix metallopeptidase 9 (MMP-9), expression of oxidized proteins, level of GAG, and expression levels of reactive oxygen species (ROS)-associated proteins, such as NADPH Oxidase (NOX) 1, NOX-2, and NOX-4, in the bladder tissue of acute IC rats (Figure 3) [163]. In this study, the transplantation of AD-MSCs was performed by intravenous (i.v.) injection. In addition, a marked upregulation of the cellular antioxidant factors, such as Glutathione peroxidase (GPx), Heme oxygenase-1 (HO-1), NAD (P)H Quinone Dehydrogenase 1) (NQO 1), and glutathione reductase (GR), was detected. Of note, melatonin possesses a potent direct and indirect antioxidant capacities as well as anti-inflammatory function, which are involved in cell membrane stabilization and cryoprotection against oxidative stress-related damage and inflammation [164,165,166,167,168]. Melatonin promotes the increased synthesis of glutathione and the production of the intrinsic antioxidant agents that quell the oxidative stress consequences [169,170,171].

In 2015, Song et al. demonstrated the therapeutic potential of hUC-MSCs in a rat model of IC, which was induced by the intravascular instillation of 0.1 M hydrochloric acid (HCl) [172]. A single injection of hUC-MSCs into the submucosal layer of the urinary bladder, one week after IC induction, significantly mitigated the IC-associated symptoms in rats. These rats exhibited a lower voiding frequency than the rats in the phosphate buffered saline (PBS)-injected group. The wingless-related integration site (WNT) signaling pathway is involved in the hUC-MSC-mediated therapeutic activity, as treatment with small molecules that inhibit WNT signaling-related genes and its downstream factors, such as EGF, insulin-like GF (IGF), and FGF, abolishes this therapeutic activity [172].

In 2016, Hirose et al. reported the potential of DPSCs in alleviating HCl-induced cystitis after injection into the bladder of female F344/NSlc rats [173]. The measurement of various cytokines and chemokines from DPSC-derived CM revealed high levels of VEGF, FGF2, and chemokines of the C-C and C-X-C chemokine families. Moreover, the authors demonstrated a marked downregulation of myeloperoxidase (MPO) and the proinflammatory IL-1β, IL-6, and TNF-α in rat bladder tissue and urine [173].

In 2018, Furuta et al. demonstrated the therapeutic potential of AD-MSCs in the alleviation of HCl-induced IC in rats [174]. After instilling 0.1 N HCl into the bladder, high nociceptive behavior, mast cell infiltration, high expression of collagen fibers (fibrosis), and upregulation of TNF-α and TGF-β were observed. Injection of AD-MSCs into the bladder wall resulted in a significant alleviation of the previously mentioned symptoms compared to the untreated control group [174].

In 2017, Xiao et al. demonstrated the beneficial effects of BM-MSCs in a protamine sulfate (PS)-induced IC rat model by modulating the TGF-β/microtubule associated protein kinase (MAPK) signaling pathway [175]. The BM-MSCs were transplanted via i.p. injection.

In 2017, Li et al. reported the protective effects of USCs against PS/lipopolysaccharide (LPS)-induced IC in rats [176]. Bladder instillation of PS followed by LPS could mimic the symptoms of chronic IC/BPS [176,177,178]. The i.v. injection of USCs in PS/LPS-induced IC rats caused a significant recovery of bladder function and marked increases in the expression levels of antioxidant proteins and anti-apoptotic proteins, such as B-cell lymphoma-2 (BCl-2), NAD (P)H quinine oxidoreductase (NQO)-1, and heme oxygenase (HO)-1 in the bladder tissue. There was a clear downregulation of inflammatory-related, apoptotic-related, oxidative stress-related, and autophagy-related markers upon injection of USCs compared to the control (untreated) group [176]. In 2019, Chung et al. screened the potential of various types of ASCs, namely USCs, ADSCs, BMSCs, and AFSCs, in the recovery of UP II-mediated bladder damage in female rats [179]. In this study, all the tested stem cells showed a clear recovery capacity against UP II-induced IC compared to the control group. Treatment with USCs resulted in an anti-inflammatory effect that was superior to that by other stem cells as shown by PCR analysis. Moreover, the authors showed that treatment with stem cells transplanted by injection into the bladder submucosa was markedly better than that via tail vein injection or transurethral instillation in terms of retaining the regenerative and anti-inflammatory potentials of the stem cells [179].

In 2018, Xie et al. reported the in vitro and in vivo effects of human umbilical cord-derived MSCs (hUC-MSCs) in IC via co-culture with TNF-α-exposed human uroepithelial cells (SV-HUC-1) and in a CYP-induced IC rat model, respectively [180]. When hUC-MSCs were injected (i.v. injection via the tail vein) one week after CYP injection in rats, the IC-related symptoms, including urine voiding, were mitigated and the pathological consequences in the bladder, such as urothelium apoptosis and mast cell infiltration, were significantly reduced. Similarly, co-culturing of hUC-MSCs with TNF-α-exposed SV-HUC-1 led to a reduction in apoptotic changes (decreases in caspase 3 cleavage) and a significant increase in the phosphorylation of p-AKT and p-mTOR proteins [180]. In 2016, Kim et al. reported the ameliorative effect of human umbilical cord-blood-derived MSCs (hUCB-MSCs) on ketamine-induced cystitis in rats [181]. Ketamine, which is a non-competitive N-methyl-D-aspartic acid (NMDA) receptor antagonist, is considered a type of recreational drug since its abuse can negatively impact bladder function. This causes IC-like lower tract symptoms, such as pain, voiding, hematuria, urgency, and frequency [182,183,184,185]. This model was established through two cycles of ketamine treatment, whereby 10-week-old female Sprague-Dawley rats were administered via the i.v. injections of ketamine for five days followed by two days off per cycle. Two days after the final ketamine dose, hUCB-MSCs were injected into the external wall or the dome of the bladder. The injection of hUCB-MSCs markedly alleviated the ketamine-induced IC in rats as significant improvements were observed in bladder function as well as in the recovery of pathological lesions such as bladder tissue fibrosis, mast cell infiltration, and apoptosis. Notably, there were marked increases in the expression levels of TGF-β signaling-associated genes and the phosphorylation of Smad2 and Smad3 proteins [181].

### 6.3. Bladder Augmentation

In addition to the previous applications described, ASCs could represent a suitable autologous cell source for bladder tissue regeneration. In 2011, Sharma et al. tested the applicability of autologous BM-MSCs in the bladder augmentation in nonhuman primates (baboon) injured during cystectomy [186]. Baboon-derived BM-MSCs transduced with a green fluorescent protein (GFP) were seeded onto scaffolds of the small intestinal submucosa and then transplanted into baboons that were subjected to cystectomy for 40–50% of the bladder. After the engraftment, the bladder capacity recovered by 28–40%, as detected by the high population of GFP cells expressing muscle markers and the proliferation marker Ki-67 [186].

In 2015, Bury et al. reported the potency of BM-MSCs seeded onto [poly(1,8-octanediol-co-citrate)] scaffolds. These stem cells not only promoted the augmentation of the rat bladder but also decreased the level of proinflammatory CD68-positive macrophages, proinflammatory cytokines (TNF-α and IL-1b), and myeloperoxidase-positive neutrophils. This occurred simultaneously with the upregulation of anti-inflammatory cytokines, namely IL-10 and IL-13 [187]. In 2015, a study by Snow-Lisy et al. showed that genetically modified MSCs via the combination of hBM-MSC with CD34-positive hematopoietic/stem progenitor cells (HSPCs) could regenerate the bladder of nude rats [188]. Additionally, various studies proved the potential of ADSCs [189,190], AD-MSCs [191], hair follicle-derived stem cells [192,193], and USCs in bladder regeneration [149,194,195]. However, the impact of these cells in IC/BPS therapy needs to be verified in further studies.

## 7. PSCs in Bladder Regeneration and IC/BPS Therapy

### 7.1. Differentiation into Bladder Cells

As we described the importance of urothelium differentiation in IC/BPS therapy earlier, the differentiation of PSCs into new bladder tissue such as urothelium is one of the crucial approaches of PSC-mediated IC/BPS therapy. In this regard, various research groups have developed novel methods for differentiating PSCs into bladder urothelium [14,196,197,198,199].

In 2009, Xie et al. demonstrated the differentiation of mouse iPSCs (miPSCs) (O9 and TT025) and mouse ESCs (mESCs) into SMCs after treatment with all-trans retinoid acid (RA) (10^−5^ mol/L) [200]. The expression of SMC-related markers (SMα-actin and SM myosin heavy chain) was detected in more than 40% of O9 miPSCs on the eighth day of differentiation, whereas TT025 miPSCs died before day 4. Variations in the expression patterns of SMC-related miPSCs and mESCs were detected. Verification of miPSC-derived SMC functionality was done by detecting contraction as well as calcium influx in response to stimuli [200]. Overall, different clones of miPSCs and mESCs responded to the same dose of RA differently. Therefore, further characterization is needed.

In 2010, Mauney et al. reported urothelium differentiation from mESCs, which were cultured on collagen matrices and exposed to different doses of RA (0.1–10 μM) [201]. RA-exposed mESCs showed decreased expression levels of Oct-4 and significantly increased mRNA expression levels of UPs. The high expression of endoderm-associated transcription factors, GATA4 and GATA6, was detected. Moreover, the transcriptional interaction of GATA4 and GATA6 on the promoter area of UP1B and UP2 that contains binding sites for the putative GATA sites was shown in electromobility shift analyses (EMSAs) [201]. In 2013, Franck et al. demonstrated the efficacy of silk-based scaffolds, containing rough, porous lamellar-like sheets that were coated with fibronectin in combination of RA treatment in the induction of mESCs and iPSCs [202].

**Figure 4:**
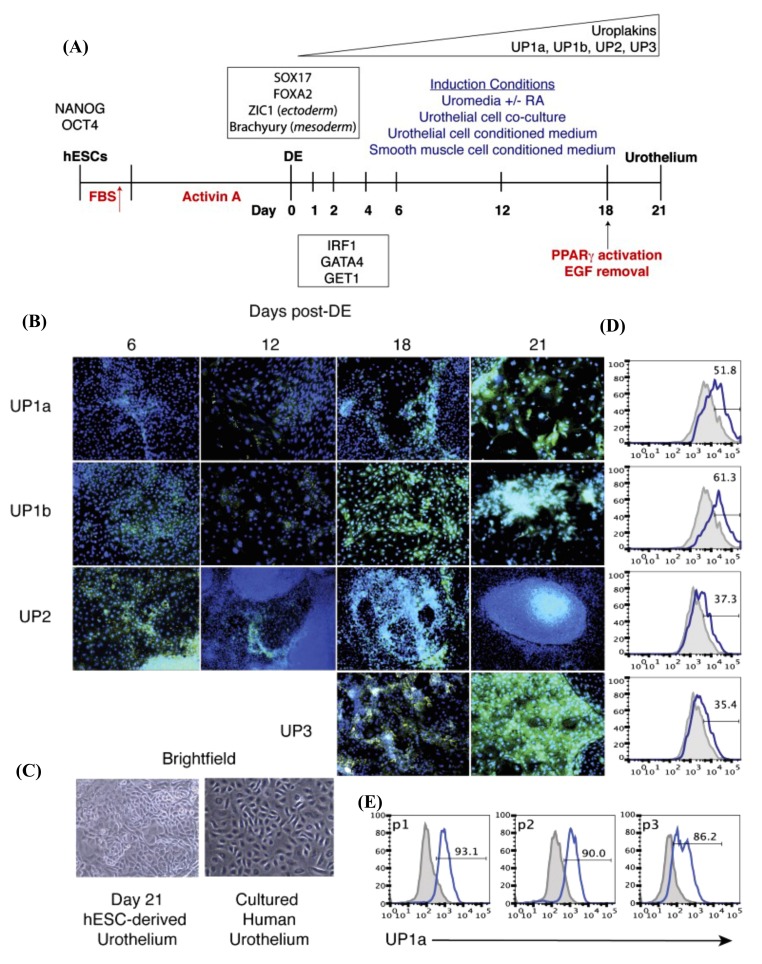

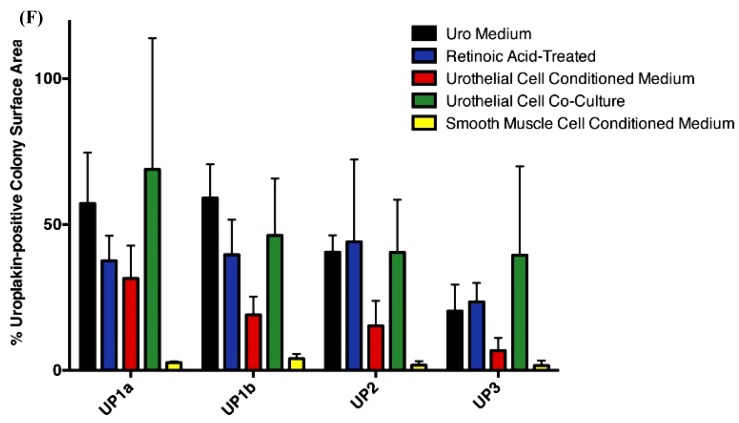
In vitro urothelium differentiation of hESCs. (**A**) Schematic diagram illustrating the protocol for urothelium differentiation of hESCs. (**B**) Intracellular cytokine flow cytometry analysis for the expression of UP subtypes (green color) on days 6, 12, 18, and 21 after DE were analyzed for the expression of all four subtypes of uroplakins (green) by intracellular cytokine flow cytometry. For nuclear counterstaining, DAPI (4′,6-diamidino-2-phenylindole; blue) was used. Magnification, 10×. (**C**) Images of cultured human urothelium and hESC-derived urothelium using bright-field microscopy on day 21 of the culture. Magnification, 10×. (**D**) Flow cytometry analysis of UP expression on day 21 of culturing. (**E**) Flow cytometry analysis for UP1a of H9-derived urothelial cultures at each passage (P 1–3). DE, definitive endoderm. (**F**) ImageJ-based quantification of the expression of each uroplakin subtype that obtained from the intracellular cytokine flow cytometry data (Figure 4B) from day 21 of culture using the five culture conditions that represented by bars of different colors. The vertical axis of Figure 4D,E represents the relative uroplakin expression amount to the colony surface area that expressed in percentage. hESCs, human embryonic stem cells. DE, definitive endoderm. hESCs, human embryonic stem cells. RA, retinoic acid. EGF, epidermal growth factor. PPARγ, peroxisome proliferator-activated receptor γ. UP, uroplakin. FBS, fetal bovine serum. These figures are reproduced from articles by Osborn et al. 2014 [14] following permission from John Wiley and Sons. In flow cytometry data, the grey histogram denotes the negative control and the solid blue line indicates the positive uroplakin staining. Up arrow (red-colored) represents the treatment of hESCs with a gradual increase in the concentration of FBS for three days that followed by Activin A treatment for nine days. Up arrow (black-colored) indicates the time point (on day 18) of adding PPARγ activator, troglitzone, and removal of EGF for induction of the terminal differentiation.

In 2014, Osborn et al. devised a protocol for the differentiation of hPSCs (hESCs and hiPSCs) into urothelium, which obviates the use of any matrices, cell contact, or adult cell signaling [14]. In this study, the cells were differentiated first into definitive endoderm (DE) characterized by high expression of the transcription factor SRY-Box Transcription Factor 17 (Sox17) and its targets Forkhead Box A1 (Foxa1) and Foxa2 [203,204]. As the cells were differentiated from hESCs, the authors tested the safety of the differentiated urothelium at passage 0 (P0) via orthotopic transplantation into non-obese diabetic (NOD)/severe combined immunodeficiency (SCID)/IL-2 receptor γ-chain knockout mice that showed negative teratoma formation. Urothelium differentiation was induced in keratinocyte basal medium supplemented with RA, EGF, bovine pituitary extract (BPE), and a low serum concentration of fetal bovine serum (FBS) (2%) (Figure 4). RA was essential for the upregulation of GATA signaling. The authors then attempted to obtain pure urothelium from hPSCs that express high levels of UPs through serial passage using specific media for the selection. Ultimately, 90% of pure urothelium was successfully obtained and passed for up to P 4. It was then cryopreserved before transplantation for the regeneration of the damaged bladder. The urothelial induction was associated with the transcription factors Interferon Regulatory Factor 1 (IRF1), Golgi to ER traffic protein 1 (GET1), and GATA-binding protein 4 (GATA4), which are correlated with the expression of UPs [14].

In 2014, Kang et al. defined culture conditions (feeder and serum free) for urothelium differentiation from hPSCs [46]. In this study, hPSCs were subjected to DE differentiation. These DE-differentiated cells were differentiated to urothelium using keratinocyte-specific serum-free medium containing RA. The differentiation was validated by the upregulation of UPIb, UPII, UPIIIa, P63, and CK-7 genes. Immunostaining was used to detect high expression of CK-8/18, UPII, and P63. Moreover, high expression of E-cadherin and ZO-1 was confirmed. The fluorescein isothiocyanate (FITC)-dextran permeability assay validated the barrier function of the hPSC-derived urothelium [46]. Thus, this defined culture method for the generation of hPSC-derived urothelium is promising for the clinical application in the regeneration of injured human bladder. In 2019, Suzuki et al. successfully differentiated hiPSCs into mature stratified bladder urothelium via treatment with a high concentration of CHIR99021, a GSK3β inhibitor, which promotes hindgut differentiation during the induction of DE [196]. Furthermore, the authors promoted terminal differentiation as well as urothelium stratification via an EGF receptor (EGFR) inhibitor, PD153035, and peroxisome proliferator-activated receptor γ (PPARγ) agonist, troglitazone. Notably, PPARγ, which is a nuclear hormone receptor, is a key factor in the terminal differentiation of urothelium and adipocytes [205,206]. Terminal differentiation was indicated by the increased expression levels of UPs, CK13, and CK20. Moreover, FGF10 treatment in a Transwell culture system further enhanced terminal differentiation and stratification. The barrier function of hiPSC-derived urothelium was shown with the permeability assay via FITC-dextran.

In 2007, Oottamasathien et al. carried out directed differentiation of mESCs into bladder tissue via specific recombination with rat embryonic bladder mesenchyme (rEBM) isolated on embryonic day 18 [207]. The mESCs were first differentiated to the DE stage as shown by the high expression of Foxa1 and Foxa2 on day 7 and day 14. Lastly, the mature urothelial cells were obtained as they showed high expression of UPs. Notably, the authors successfully figured out the accurate rEBM to mESCs ratio that avoids teratoma formation after several experimental trials [207]. In 2008, the same research team tried to analyze the temporo-spatial expression of the DE stage and mature urothelial cell-associated markers after engraftment with the recombination of rEBM and mESCs under the kidney capsule of the mouse [208]. For this purpose, the grafts were collected at various time points (day 16, day 18, day 21, day 35, and day 42) and then the expression levels of the specific markers were estimated at each time point. The expression of UPs was detected on day 16, which correlated with the loss of Foxa2 expression. In contrast, the expression of Foxa1 was detected at all time points. The androgen receptor was first detected in the stroma at day 16 in the nucleus of the urothelial cells on day 21. No expression of the androgen receptor was detected on day 42 [208]. These studies represent the expression of specific markers at various stages of the bladder development.

In 2008, Kinebuchiet al. screened the efficacy of four media in the induction of mESCs into urinary tract tissue [209]. This research team seeded embryoid bodies (EBs) derived from mESCs onto one layer of collagen membranes and then exposed them to four culture media, including medium 1, mixture of keratinocyte serum-free medium (KSFM) and Medium 199, medium 2, mixture of 3T3 fibroblast-derived CM and KSFM, medium 3, control medium (EB formation medium), and medium 4, control medium composed of KSFM. After 28 days of culturing, the cells were transplanted into a nude mouse. The culture conditions using media 1 and 2 resulted in the formation of a structure with four layers composed of a stratified epithelium, a layer of smooth muscle cells, and loose connective tissue in the submucosa [209]. UP positive urothelium-like cells were detected only after the culture with medium 2. In contrast, culturing with media 3 and 4 resulted in necrotic changes. However, further studies are needed to reveal the mechanisms of media 2-mediated formation of urothelium for its use and application in bladder regeneration.

### 7.2. Direct Transplantation

PSCs as well as PSC-derived MSCs have therapeutic potential. The therapeutic potential of PSC-MSCs over primary or tissue-derived MSCs in tissue regeneration has been demonstrated previously [210,211,212]. For example, Lee et al. showed that MSCs derived from hESCs (M-MSCs) were superior to BM-MSCs in treating IC in rats that had chemically induced IC from ketamine hydrochloride [213]. Female Sprague-Dawley rats (10-week old) were subjected to alternating i.v. and i.p. injections of ketamine hydrochloride twice per week for 12 weeks. Subsequently, M-MSCs were injected into the anterior bladder wall or bladder dome one week after the last ketamine injection. One week later, the therapeutic efficacy of the injected cells was evaluated. The authors reported significant recovery from ketamine-associated IC symptoms including, voiding, tissue fibrosis, apoptosis, and high mast cell infiltration. Notably, injection of low concentrations of M-MSCs (1 × 10^5^ cells) led to a marked improvement in bladder function compared to treatment with BM-MSCs at the same cell number, which did not improve the IC symptoms [213]. Thus, the potent anti-fibrotic activity of M-MSCs or MSCs is the main attribute in the treatment of the ketamine-induced IC rat model.

In 2017, Kim et al. compared the therapeutic potential of M-MSCs with tissue-derived MSCs, such as BM-MSCs in the HCl-induced IC/BPS rat model. M-MSCs or BM-MSCs were locally injected into the external layer of the dome and anterior wall of the bladder one week after HCl instillation [214]. M-MSCs significantly improved the symptoms of HCl-induced IC/BPS, including urine voiding and histopathology lesions, and were markedly more robust than BM-MSCs injections with 10-fold fewer injected cells than BM-MSCs [214]. No significant negative consequences, such as immune rejection, tumorigenicity, and abnormal growth until more than 12 months after the cell injection, were detectable. Moreover, the continual monitoring of the transplanted GFP-expressing M-MSCs in the rat injured bladder could be tracked for longer than six months using longitudinal intravital confocal fluorescence imaging. Notably, these transplanted cells were observed as E-cadherin positive urothelium and vimentin positive pericytes or stroma cells. This potent therapeutic effect is ascribed to Wnt and IGF signaling [214].

**Figure 5:**
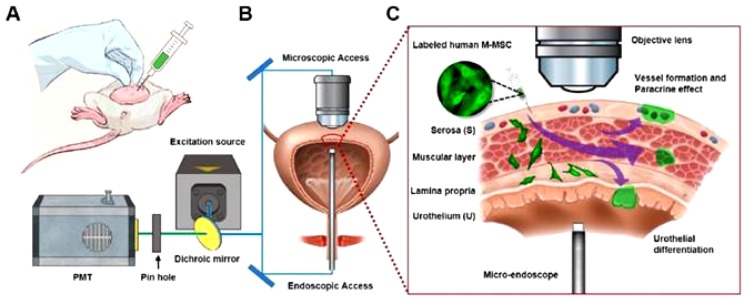
Diagram illustrating in vivo real-time tracking of transplanted M-MSCs in an IC/BPS rat by longitudinal intravital confocal fluorescence imaging. (**A**) Diagram illustrating the transplantation of M-MSCs via injection into the bladder wall. (**B**) Scheme displaying the process of monitoring the distribution, migration, and integration of the injected M-MSCs using the confocal imaging of the external bladder wall and micro-endoscopy of the internal bladder wall. (**C**) Representative diagram describing the migration of M-MSCs from the injection site (the serosa) to the lamina propria and urothelium. At first, M-MSCs differentiate into the urothelium to restore the damaged urothelium and then assimilate into blood vessel-like structures. This figure is reproduced from articles by Ryu et al. 2018 [177]. This is an open access article distributed under the terms of the Creative Commons Attribution (CC BY-NC) license (https://creativecommons.org/licenses/by-nc/4.0/).

In 2018, Ryu et al. performed in vivo real-time tracking of M-MSCs, after their transplantation into a chronic IC/BPS rat model, using longitudinal intravital confocal fluorescence imaging and micro-cystoscopy imaging [177]. In this study, the IC/BPS rat model was induced via bladder instillation of PS that was followed by LPS once per week for five weeks. One week after the last PS/LPS instillation, increasing quantities of M-MSCs were injected into the outer layer of the bladder (0.1, 0.25, 0.5, and 1 × 10^6^ cells). The significant improvement in the bladder functions including voiding and the recovery of the histopathological lesions after M-MSCs transplantation was clearly higher than that shown by BM-MSCs. The combined longitudinal intravital confocal fluorescence imaging and microcystoscopy detected the engraftment of the transplanted cells after their differentiation into several cell types with the gradual assimilation into a perivascular-like structure up to one-month post-transplantation (Figure 5) [177]. PS/LPS instillation resulted in the significant downregulation of WNT family-related genes, namely Wnt2b, Wnt4, Wnt5a, Wnt8a, Wnt8b, and Wnt10a, and the downstream GF, Smoothened (Smo), a transducer of sonic hedgehog (SHH) signaling, such as IGF2, FGF9, and Notch homolog 1 (Notch 1) [177].

## 8. Conclusions and Future Prospects

In this review, we provided a brief overview of IC/BP etiology, pathophysiology, diagnosis, and therapeutic approaches based on the recent literature. IC/BPS is a chronic syndrome in which a wide range of factors are implicated including disruption in the bladder urothelium function, mast cell infiltration, autoimmune diseases, UTI, inflammatory diseases, and disturbance in urothelium signaling. These etiological factors lead to urothelium damage and leakage of urine solutes, especially potassium salts into the lamina propria that result in hypersensitivity of the neurons and, ultimately, pain. Autoimmunity is implicated in the pathogenicity of IC/BPS, which motivates scientists to apply experimental autoimmune cystitis (EAC) animal models to study autoimmune-associated IC/BPS pathogenicity that may not have inflammation [215,216,217]. In contrast, the induction of IC/BPS in vivo is performed by the intravesical instillation of noxious chemical agents such as CYP, HCL, ketamine, PS/LPS, and hydrogen peroxide, which induce an acute form of IC/BPS accompanied by inflammation [218,219,220]. Thus, selection of a suitable IC/BPS model should be considered. There are various approaches for IC/BPS therapy. Unfortunately, most of those are focused on alleviating the pain and inflammation. Moreover, no efficient therapeutic agent has been discovered as a curative therapy. Accordingly, our review discusses the possible approaches for applying stem cells (ASCs and PSCs) in bladder regeneration and IC/BPS therapy, which are summarized in Table 1. Many preclinical studies demonstrate biosafety and the potential of stem cells to treat bladder diseases [221]. As described above, the therapeutic potentials of ASCs and PSCs in bladder regeneration are attributed to their ability to differentiate into key bladder cells, such as urothelium and SMCs. Moreover, the direct transplantation of stem cells via various routes (i.v., i.p., and injection into the bladder wall) showed robust therapeutic actions. Notably, several mechanisms are involved in stem cell-mediated bladder regeneration such as activation of key signaling pathways including AKT, mTOR, ERK, and Wnt-GSK3β/β-catenin. Moreover, stem cell-derived CM rich in GF as well as the combination of a low dose of EGF and stem cells are involved in urothelium differentiation and bladder repair. Therefore, further formulation of GF and stem cells using low doses of GF and short exposure time need to be characterized in the future to obtain superior effects. However, the molecular mechanisms of stem cells during bladder regeneration and IC/BPS therapy need to be characterized in future studies. Moreover, the activities of ASCs and PSCs in vitro need to be verified in vivo as well. Future studies that address the controversies in the literature about the route of transplantation and safety of stem cells are essential for further clinical application. The research data using IC/BPS animal models should be meticulously scrutinized before further clinical trials since animal models have limitations in emulating human diseases. Alternatively, the generation of bladder organoids from human stem cells should be considered in the future for studying the pathogenicity of IC/BPS and drug screening [222,223].

Several reports display the wide-range therapeutic application of stem cell-derived exosomes via various mechanisms [224,225]. The therapeutic impact of exosomes isolated from ASCs and PSCs in the therapy of IC/BPS is strongly recommended for future research. We believe that a better understanding of the therapeutic mechanisms of stem cells in correlation with the pathophysiology of IC/BPS is crucial for their clinical application.

## Figures and Tables

**Table 1:** Table summarizing the implications of adult stem cells (ASCs) and pluripotent stem cells (PSCs) in bladder regeneration and interstitial cystitis/bladder pain syndrome (IC/BPS) therapy.

Stem Cell Type	Cell Line Name	Regeneration Method and In Vitro Verifications	In Vivo Model	References
**ASCs**	**BM-MSCs**	- Urothelium and SMCs differentiations using urothelial cell-derived CM or co-culture with urothelial cells using Transwell plates. - High expression of UP1a, CK-7, and CK-13 in differentiated cells. - CM showed superior action than co-culture system. - High content of PDGF-BB, TGF-β1, and VEGF in the CM.	n.a.	[144]
		- Urothelial differentiation through co-culture with neonatal urothelial cells. - High expression of urothelial-related keratin after two weeks of co-culturing. - Detection of microstructural epithelial cell-like features with the electron microscopy.	n.a.	[145]
		- Transplantation was carried out through the injection of BrdU-labeled BMSCs (2 × 10^5^/mL) into the tunica muscularis vesicae urinariae.	- PS-induced IC rat model. - BM-MSCs injection improved the urine excretion kinetics and recovered the pathological lesions. - TGF-β/MAPK signaling pathway-dependent repair mechanism.	[175]
	**hUC-MSCs**	- Urothelial differentiation by co-culturing with urothelial cell-derived CM containing 20 ng/mL exogenous EGF. - High expression of UPs and CK.	n.a.	[146]
		- Single injection of hUC-MSCs into the submucosal layer of the urinary one week after HCl injection.	- HCl-induced IC/BPS female rat model. - Marked improvement of IC/BPS-related symptoms. - WNT signaling is involved in hUC-MSC-mediated IC/BPS recovery in the rat.	[172]
		- i.v. injection of hUC-MSCs (1 × 10^6^ cells) in CYP-induced chronic IC/BPS rat model. - In vitro verification via the co-culture of UC-MSCs with TNF-α-treated SV-HUC-1. This co-culture led to decreases in apoptotic changes (decrease in caspase 3 cleavage) and increases in the phosphorylation of AKT and mTOR.	- CYP-induced chronic IC/BPS rat model. - Significant recovery of the bladder function and the pathological changes in bladder tissue.	[180]
		- Single i.v. of UCB-MSCs at a dose of 1 × 10^6^ cells.	- Ketamine-induced cystitis in rat. - UCB-MSC injection led to a significant improvement in bladder functions and pathological changes. - Upregulation of TGF-β signaling-associated genes and high phosphorylation of Smad2 and Smad3 proteins.	[181]
	**hAFSCs**	- Urothelial differentiation through co-culturing with bladder cancer-derived CM. - Low expression of pluripotency markers and increase in urothelial markers expression, such as UPII, CK8, and FGF10. - Increase in the expression of MuSK, Tie-1, and EphA4 receptor tyrosine kinases and the downregulation of EphA7 and FGF R1 kinases.	n.a.	[157]
		- Urothelial differentiation through co-culturing with the immortalized bladder cancer cell lines. - FGF10-mediated differentiation mechanism. - High mRNA and protein expression levels of UP II, UP III, and CK-8. - MEK inhibitor, U0126, inhibited urothelium differentiation.	n.a.	[158]
	**DPSCs**	- SMCs differentiation using bladder SMC-derived CM containing TGF-*β*1. - Upregulation of SMC-associated genes and proteins (α-SMA, desmin, and calponin). - Wnt-GSK3β/β-catenin pathway is involved in differentiation.	n.a.	[160,161]
		- Cell injection into the bladder (2.0 × 10^6^ cells in PBS). - DPSC-derived CM contains high level of FGF-2, VEGF, C-C and C-X-C chemokine families.	- HCl-induced cystitis in female F344/NSlc rat model. - Reduction in MPO and proinflammatory cytokines (IL-1β, IL-6, and TNF-α) in the bladder tissue and urine after DSPSC injection.	[173]
	**USCs**	- Urothelial differentiation induction using urothelial-derived CM. - Upregulation of urothelial markers, UP II and UP1a, tight junction markers, Cingulin, E-cadherin, ZO-1, and ZO-2, and the epithelial markers (AE1/AE3 and CK20). - Differentiated urothelium possess barrier function.	n.a.	[153]
		- Interstitial cell differentiation via transfection of USCs with lentiviral vectors encoding SCF, c-kit, AZI2 gene, and HCN4. - Differentiated cells showed high expression of the c-kit after one week.	n.a.	[154]
		- i.v. injection of USCs (1.2 × 10^6^ cells).	- PS/LPS-mediated chronic IC/BPS rat model. - Marked recovery of the bladder functions in USC-injected rat. - Upregulation of the antioxidant and the anti-apoptotic proteins in the bladder tissue. - Downregulation of oxidative stress-related and autophagy-related markers.	[176]
	**ADSCs**	- Urothelial differentiation induction using urothelial-derived CM. - Polygonal epithelium-like morphology. - Upregulation of UP2 and CK-18 and downregulation of vimentin. - High content of PDGF-BB and VEGF in CM is implicated in differentiation induction.	n.a.	[162]
		- The combination of i.v. injection of autologous AD-MSCs (1.2 × 10^6^ cells) and i.p. injection of melatonin.	- CYP-induced acute IC/BPS. - Marked decrease in urine volume, urine albumin, hematuria, and proteinuria. - Decreased tissue fibrosis (low collagen deposition). - Upregulation of the cellular antioxidant markers, HO-1 and NQO. - Downregulation of the inflammatory markers in tissue and urine.	[163]
		- Injection of AD-MSCs (1 × 10^6^ cells in PBS) into the anterior and posterior bladder walls during injection of the second dose of HCl.	- HCl-induced IC/BPS rat model. - Significant decrease of nociceptive behavior, mast cell infiltration, and bladder fibrosis. - Downregulation of TNF-α and TGF-β.	[174]
**PSCs**	**ESCs**	- Urothelium and SMCs differentiation of mESCs via culture on collagen matrices and RA treatment in a dose-dependent manner. - Downregulation of pluripotency markers and significant increase in the mRNA level of UPs. - Upregulation of the transcription factors, GATA4 and GATA6, associated with UP1B and UP2 activation.	n.a.	[201]
		- Urothelium differentiation of mESCs through the recombination with rEBM. - High expression of UPs.	- Engraftment of the recombinant mESCs and rEBM under a kidney capsule of mouse. - Harvesting of the grafts at various time points. - At day 16: high expression of UPs, androgen receptor expression, and loss of the expression of Foxa2.	[207,208]
		- Differentiation of mESCs into bladder cells. - Seeding of mESC-derived EBs on mono-layered collagen membrane using various KSFM-based differentiation media. - High expression of SMC-related markers (αSMA and hl-calponin, and SM-MHC), UPII, and Pax-2.	- On day 28 after the in vitro culture on collagen and application of several differentiation media, the cultured tissues were transplanted subcutaneously into the back of male nude mice (BALB/c nu/nu). - The transplantation of tissue cultures using KSFM and 3T3-CM resulted in the formation of layered tissue like that of the urinary tract.	[209]
		- hESC-differentiated DE was directed toward urothelium differentiation using keratinocyte basal medium containing RA, EGF, and BPE and 2% FBS. - RA is implicated in GATA signaling activation. - Pure urothelium (up to 90%) was obtained after several passages and selection using specific media. - Co-expression of IRF1, GET1, and GATA4 with UPs expression.	n.a.	[14]
		- Differentiation of hESC-DE into urothelium using KSFM supplemented with RA. - Upregulation of UPIb, UPII, UPIIIa, P63, CK-8/18, CK-7, E-cadherin, and ZO-1. - hESC-derived urothelium possesses a barrier function.	n.a.	[46]
		- Direct transplantation of M-MSCs through the injection of cells in a dose-dependent manner (0.25, 0.5, and 1 × 10^6^ cells) into an anterior bladder wall or bladder dome.	- ketamine-induced IC/BPS rat model. - Significant improvement in bladder function with an effect superior to that of BM-MSCs when injected at a low concentration (1 × 10^5^ cells).	[213]
		- Injection of M-MSCs (1 × 10^6^ cells) into the outer layer of the anterior wall and dome of the bladder.	- HCl-induced IC/BPS rat model. - Significant restoration of bladder function and pathological changes in bladder tissue. - No tumor formation or immune rejection was detected over a long period post-injection (12 months). - Injected cells were differentiated into several cell types and progressively assimilated into a vascular-like structure. - Wnt-mediated and IGF-mediated repair mechanism.	[214]
		- Dose-dependent injection of M-MSCs (0.1, 0.25, 0.5, and 1 × 10^6^ cells) into the outer layer of the bladder (In vivo urothelium differentiation of M-MSCs).	- PS/LPS-induced IC/BPS rat model. - Marked recovery of the bladder function and pathological consequences in the tissue. - Stability: Long-term maintenance of the therapeutic capacity of the transplant. - Differentiation of the transplant into multiple cell types and integration into a perivascular-like structure (up to a month) post-injection).	[177]
	**hiPSCs**	- Urothelium differentiation of hiPSCs using a high dose of GSK3β inhibitor CHIR99021 during DE differentiation. - Combination of EGFR inhibitor, PD153035 and PPARγ agonist, Troglitazone enhanced terminal urothelium differentiation. - High expression of UPs, CK13, and CK20. - FGF10 and Transwell culture system promoted further stratification. -Barrier function.	n.a.	[196]

BM-MSC, bone marrow-derived mesenchymal stem cell. SMCs, smooth muscle cells. CM, conditioned media. UP, uroplakin. PDGF-BB, platelet-derived growth factor-homodimer polypeptide of B chain. TGF-β1, transforming growth factor beta 1. VEGF, vascular endothelial growth factor. BrdU, Bromodeoxyuridine. PS, protamine sulfate. LPS, lipopolysaccharide. HCL, hydrochloric acid. UC-MSC, umbilical cord-derived mesenchymal stem cell. UCB, umbilical cord blood. IC/BPS, interstitial cystitis/bladder pain syndrome. MuSK, muscle-specific kinase. Tie-1, Tyrosine kinase with immunoglobulin-like and EGF-like domains 1. EphA4, Ephrin type-A4. MPO, myeloperoxidase. TNF, tumor necrosis factor. IL, interleukin. PPARγ, peroxisome proliferator-activated receptor γ. SV-HUC, SV40 immortalized human uroepithelial cell. mTOR, mammalian target of rapamycin. AKT, protein kinase B. CK, cytokeratin. FGF, fibroblast growth factor. FBS, fetal bovine serum. i.v., intravenous. i.p., intraperitoneal. MEK, mitogen-activated protein/extracellular signal-regulated kinase. α-SMA, α-smooth muscle actin. GSK, glycogen synthase kinase. PBS, phosphate-buffered saline. DPSC, dental pulp stem cell. Pax-2, Paired box gene 2. AZI2, the 5-azacytidine-induced 2. HCN4, hyperpolarization-activated cyclic nucleotide gated potassium channel 4. SCF, stem cell factor. AD-MSC, adipose-derived mesenchymal stem cell. rEBM, rat embryonic bladder mesenchyme RA, retinoic acid. rEMB, rat embryonic bladder mesenchyme. SM-MHC, smooth muscle myosin heavy chain. DE, definitive endoderm. Wnt, wingless-related integration site. KSFM, keratinocyte serum-free medium. CYP, cyclophosphamide. EGF, epidermal growth factor. IRF1, Interferon Regulatory Factor 1. ZO-1, Zona Occludens 1. IGF, insulin-like GF. Foxa1, Forkhead Box A1. GET1, Golgi to ER traffic protein 1. M-MSC, hESC-derived MSCs. PPARγ, peroxisome proliferator-activated receptor γ. BPE, bovine pituitary extract. EGFR, epidermal growth factor receptor. USC, urine-derived stem cell. ADSC, adipose derived mesenchymal stem cell. ESCs, embryonic stem cells. AFSC, amniotic fluid-derived stem cell. hiPSC, human induced pluripotent stem cell. n.a., not determined.
